# Distribution of Cannabinoid Receptors in Keratinocytes of Healthy Dogs and Dogs With Atopic Dermatitis

**DOI:** 10.3389/fvets.2022.915896

**Published:** 2022-07-08

**Authors:** Roberto Chiocchetti, Margherita De Silva, Francesca Aspidi, Rodrigo Zamith Cunha, Francesca Gobbo, Claudio Tagliavia, Giuseppe Sarli, Maria Morini

**Affiliations:** Department of Veterinary Medical Sciences (UNI EN ISO 9001:2008), University of Bologna, Bologna, Italy

**Keywords:** cannabidiol, GPR55, immunohistochemistry, palmitoylethanolamide, PPARα, 5-HT1A receptor, TRPA1, TRPV1

## Abstract

It is commonly accepted that some form of skin barrier dysfunction is present in canine atopic dermatitis (AD), one of the most common cutaneous pruritic inflammatory diseases of dogs. The impaired skin barrier function facilitates the penetration of allergens and subsequently stronger sensitization responses. The role of the endocannabinoid system (ECS) in the physiology and pathology of the skin is becoming increasingly established. It has been demonstrated that cannabinoid receptors are expressed in healthy and diseased skin and, based on current knowledge, it could be stated that cannabinoids are important mediators in the skin. The present study has been designed to immunohistochemically investigate the expression of the cannabinoid receptors type 1 (CB1R) and 2 (CB2R) and the cannabinoid-related receptors G protein-coupled receptor 55 (GPR55), transient receptor potential vanilloid 1 (TRPV1) and ankyrin 1 (TRPA1), peroxisome proliferator-activated receptors alpha (PPARα), and serotoninergic receptor 1a (5-HT1aR) in keratinocytes of healthy dogs and of dogs with AD. Samples of skin tissues were collected from 7 healthy controls (CTRL-dogs) and from 8 dogs with AD (AD-dogs). The tissue samples were processed using an immunofluorescence assay with commercially available antibodies, and the immunolabelling of the receptors studied was quantitatively evaluated. The keratinocytes of the CTRL- and the AD-dogs showed immunoreactivity for all the receptors investigated with a significant upregulation of CB2R, TRPA1, and 5-HT1aR in the epidermis of the AD-dogs. The presence of cannabinoid and cannabinoid-related receptors in healthy keratinocytes suggested the possible role of the ECS in canine epidermal homeostasis while their overexpression in the inflamed tissues of the AD-dogs suggested the involvement of the ECS in the pathogenesis of this disease, having a possible role in the related skin inflammation and itching. Based on the present findings, the ECS could be considered a potential therapeutic target for dogs with AD.

## Introduction

Canine atopic dermatitis (AD) is one of the most common canine cutaneous inflammatory and pruritic diseases (1) in which allergens sensitize skin by aerogenous or alimentary routes (2), or an impaired skin barrier function facilitates the penetration of allergens and the subsequent sensitization responses (3–5). It is presumed, however, that allergen exposure occurs through the skin in which antigens trapped by Langerhans cells are presented to γ/δ T cells (2). Several factors, such as exposure to pollutants, changes in dietary habits, stress, genetic factors and cutaneous infections, appear to contribute to the skin inflammation and pruritus.

Several cell types, such as keratinocytes, Langerhans cells (LCs), dendritic cells (DCs), mast cells (MCs), basophils, eosinophils, monocytes and activated T cells may display some abnormality in AD. Nevertheless, the key effector cells in AD are thought to be keratinocytes, LCs/DCs, and Th2, Th17 and CD4+/CD25+ regulatory T cells (6–8).

The pivotal role of keratinocytes (and immune cells) in the AD pathogenesis has been demonstrated not only in humans and rodents (9), but also in dogs (4). Keratinocytes respond to antigens and pathogens by virtue of their wide range of surface receptors, and produce several inflammatory mediators which could affect neighboring cells (paracrine), reach the circulation and affect distant sites (endocrine), or modulate their own functions (autocrine) (6). The granulocyte-macrophage colony-stimulating factor (GM-CSF) produced by keratinocytes, leading to the proliferation of granulocytes and macrophages (10), contributes to the maintenance of chronic inflammation in atopic patients.

A Th2-polarized lymphocyte response (11–13), in which keratinocytes seem to play a determinant role, occurs during the correlated (atopic) hypersensitivity reaction. In fact, in a barrier-disrupted epidermis, keratinocytes produce large amounts of thymic stromal lymphopoietin (TSLP) and other cytokines (IL-25 and IL-33) which lead to Th2 immune deviation (14). To aggravate the situation, the transcriptional effect of Th2 type (IL-4 and IL-13) and Th17 (IL-17) cytokines alters the integrity of the epidermis by reducing the production of filaggrin in keratinocytes which additionally compromises the function of the skin barrier (15, 16). In general, the extent of the skin barrier dysfunction correlates with the degree of inflammation within AD lesions (17).

Of the cytokines released by Th2 cells, the role of IL-31 consists of mediating the inflammatory responses and stimulating itching by means of the activation of IL-31 receptor A (IL-31RA), expressed not only on human and murine dorsal root ganglia neurons, but also on keratinocytes and various innate immune cells (18, 19). Interleukin-31 affects keratinocyte differentiation, downregulates the filaggrin expression and upregulates the expression levels of pro-inflammatory cytokines (20). The administration of IL-31 also evokes the scratching behavior of dogs (21), and the anti-canine IL-31 antibody (lokivetmab) significantly inhibits scratching in dogs with AD (22, 23).

Keratinocytes of patients with AD may produce and secrete chemical substances such as neurotrophins, adenosine triphosphate (ATP), and β-endorphins which may be related to the hyperinnervation of the AD lesions (24, 25).

Therefore, it is clear that keratinocytes, and not just inflammatory cells and the immune system, must be taken into consideration as a therapeutic target in AD. One of the potential alternative therapeutic targets to be investigated during AD could be the endocannabinoid system (ECS) which has been shown to be involved in the regulation of the biological processes of the skin epidermis (26). The ECS is composed of endogenous ligands anandamide (AEA) and 2-arachidonoyl glycerol, cannabinoid receptors 1 and 2 (CB1R and CB2R), and enzymes aimed at degrading and recycling ligands (27–29). Human keratinocytes produce endocannabinoids and express CB1R and CB2R, as well as metabolic enzymes (30, 31). The ECS also includes endocannabinoid-like mediators as well as other cannabinoid-related receptors, such as the transient receptor potential (TRP) channels, the nuclear peroxisome proliferator-activated receptors (PPARs), the G protein-coupled receptors (GPRs), and the serotonin receptors (27, 32, 33).

Palmitoylethanolamide (PEA), a lipid mediator which is structurally related to anandamide, is used in veterinary and human medicine for its anti-neuroinflammatory, antipruritic, analgesic and neuroprotective properties (34, 35).

Cannabinoid and cannabinoid-related receptors can also be activated by exogenous phytocannabinoid compounds derived from *Cannabis sativa*, specifically, Δ-9-tetrahydrocannabinol (THC) and cannabidiol (CBD) (26) and by synthetic cannabinoids (36).

The ECS contributes to the homeostasis of various organs, and its dysregulation seems to be associated with several pathological conditions (28, 37, 38).

It has been shown that PEA and CBD could prove useful in dogs with AD (39). Despite this promising clinical trial, there are still only a few studies dedicated to the histological localization of cannabinoid receptors in the canine epidermis (40). Therefore, the aim of the present study was to improve the histological knowledge regarding the expression of cannabinoid and cannabinoid-related receptors in the keratinocytes of the healthy controls (CTRL-dogs) and of dogs with AD (AD-dogs). In particular, the expression of CB1R, CB2R, GPR55, TRP vanilloid 1 (TRPV1) and ankyrin 1 (TRPA1), PPAR alpha (PPARα), and serotonin receptor 5-HT1aR was investigated using immunohistochemistry.

The results of the present study could lead to future pre-clinical and clinical trials dealing with molecules which are active on the epidermis which, in turn, could improve the symptoms of dogs with AD.

## Materials and Methods

### Animals

#### Inclusion Criteria

Atopic dermatitis is a clinical diagnosis which requires the exclusion of the other more common hypersensitivity reactions: flea bite, allergic dermatitis and adverse food reaction. Therefore, only tissues belonging to those client-owned dogs which were identified as AD-dogs were used in this study with the application of Favrot's criteria (41). Cutaneous samples were collected from 8 AD-dogs (Table 1) and 7 CTRL-dogs on which any treatment had been made in the previous 6 months (Table 2). Written client consent was obtained prior to enrollment of all cases.

**Table 1:** Clinical data of the dogs with atopic dermatitis (AD) enrolled in the present study.

**Dogs**	**Breed**	**Sex**	**Age**	**Pruritus visual analog scale (PVAS) (42)** **skin area**
AD 1	Jack Russell	F^S^	7 yr	PVAS: 9/10 Groin
AD 2	French Bulldog	F	3 yr	PVAS: 8/10 Axilla (right and left)
AD 3	Cavalier King Charles Spaniel	M	8 yr	PVAS: 8/10 Groin
AD 4	Mixed breed	M	11 yr	PVAS: 6/10 Groin
AD 5	Akita Inu	M	4 yr	PVAS: 8/10 Axilla
AD 6	Golden Retriever	M	8 yr	PVAS: 9/10 Groin
AD 7	American Staffordshire Terrier	M	4 yr	PVAS: 8/10 Axilla
AD 8	French Bulldog	F	3 yr	PVAS: 8/10 Groin

*F, female; F^s^, spayed female; M, male; yr, years*.

**Table 2:** Clinical data of the control dogs (CTRL) enrolled in the present study.

**Dogs**	**Breed**	**Sex**	**Age**	**Cause of surgery** **skin area**
CTRL 1	Springer Spaniel	F	5 yr	Ovariectomy Groin
CTRL 2	German Shepherd	F	6 mo	Corrective orthopedic surgery Groin
CTRL 3	Mixed breed	F^s^	10 yr	Bite wound Axilla
CTRL 4	Mixed breed	F	10 mo	Ovariectomy Groin
CTRL 5	German Shepherd	M	5 yr	Orchiectomy Groin
CTRL 6	Golden Retriever	F^s^	2 yr	Bite wound Axilla
CTRL 7	Lagotto Romagnolo	M	3 yr	Foreign body in the skin Groin

*F, female; F^s^, spayed female; M, male; mo, months; yr, years*.

#### Sample Collection and Processing

In the AD-dogs, a biopsy sample of skin lesions located in the ventral abdominal or axillary areas (Table 1) was collected using a sterile 8 mm biopsy punch. Sampling was performed under local (2% lidocaine) anesthesia, using the same protocol for all dogs. In the CTRL-dogs, a tissue sample was collected from anesthetized subjects during routine or specific surgery, from different cutaneous locations (as indicated in Table 2) (Authorization no. 1303/2021). The tissues from the CTRL- and the AD-dogs were processed to obtain cryosections.

#### Cryosections

The samples were fixed overnight in 4% paraformaldehyde in 0.1 M sodium phosphate buffer (pH 7.0) at +4°C. After being washed in phosphate-buffered saline (PBS 0.15 M NaCl in 0.01 M sodium phosphate buffer, pH 7.2), the tissues were immersed in PBS plus sodium azide 0.1% for 48 h (+4°C) and were then preserved in PBS–sodium azide 0.1% plus sucrose 30% (+4°C). All the samples were subsequently frozen in liquid nitrogen, and 14 μm-thick cryosections were obtained. The cryosections were hydrated in PBS and processed for histology and immunostaining.

### Histopathology

The cryosections were hydrated in PBS for 10 mins and processed for histological staining with hematoxylin and eosin (H&E) following standard procedures. The sections were evaluated following the criteria of Gross et al. (43). Images were acquired using an optical microscope (Eclipse E600; Nikon, Shinjuku, Japan) equipped with a USB 3.0 camera series “33” Imaging Source (cat. No. DFK 33UX264; Bremen, Germany).

### Immunofluorescence

The cryosections were hydrated in PBS and processed for immunostaining. To block non-specific bindings, the sections were incubated in a solution containing 20% normal donkey serum (Colorado Serum Co., Denver, CO, USA), 0.5% Triton X-100 (Sigma Aldrich, Milan, Italy, Europe) and bovine serum albumin (1%) in PBS for 1 h at room temperature (RT). The sections were incubated in a humid chamber overnight at RT with the antibodies directed against the cannabinoid and cannabinoid-related receptors (single immunostaining) (Table 3) diluted in 1.8% NaCl in 0.01M PBS containing 0.1% sodium azide. After washing in PBS (3 × 10 mins), the sections were incubated for 1 h at RT in a humid chamber with the secondary antibody (donkey anti-rabbit 488, A-21206, dilution 1:500) (Thermo Fisher Waltham, MA, USA) diluted in PBS. The cryosections were then washed in PBS (3 × 10 min) and mounted in buffered glycerol at pH 8.6 with 4′,6-diamidino-2-phenylindole– DAPI (Santa Cruz Biotechnology, Santa Cruz, CA, USA).

**Table 3:** Primary antibodies used in the study.

**Primary antibody**	**Host**	**Code**	**Dilution**	**Source**
CB1R	Rabbit	Orb10430	1:200	Byorbit
CB1R	Rabbit	ab23703	1:100	Abcam
CB2R	Rabbit	ab45942	1:200	Abcam
GPR55	Rabbit	NB110-55498	1:100	Novus Biol.
PPARα	Rabbit	NB600-636	1:200	Novus Biol.
5-HT1a receptor	Rabbit	ab85615	1:100	Abcam
TRPA1	Rabbit	ab58844	1:200	Abcam
TRPV1	Rabbit	ACC-030	1:200	Alomone

*Primary antibody suppliers: Abcam, Cambridge, UK; Alomone, Jerusalem, Israel; Biorbyt Ltd., Cambridge, UK; Novus Biologicals, Littleton, CO, USA*.

### Fluorescence Microscopy

The slides were observed using a Nikon Eclipse Ni microscope equipped with the appropriate filter cubes to differentiate the fluorochrome employed. Images were recorded using a Nikon DS-Qi1Nc digital camera and NIS elements software BR 4.20.01 (Nikon Instruments Europe BV, Amsterdam, Netherlands). The figure panels were prepared using Corel Draw (Corel Photo Paint and Corel Draw, Ottawa, ON, Canada).

The immunoreactivity of the antibodies was evaluated by an expert pathologist and its distribution in the epidermis and the cellular localization (nuclear, membranous, cytoplasmic) were reported. The intensity of the expression was evalutated semiquantitatively in pictures acquired using the same exposure times, as faint, moderate and bright.

### Specificity of the Primary Antibodies

The specificities of the rabbit anti-CB1R (Orb10430), -CB2R (ab45942), -PPARα, and -GPR55 antibodies were tested on dog tissues using Western blot (Wb) analysis (44, 45). The specificity of the other anti-CB1R antibody (ab23703) used in the present study has already been tested using the absorption test on dog tissues (40). In addition, the amino acid sequences (MSVSTDTSAEAL) of the immunogens (of human origin) used to obtain the anti-CB1R antibody (ab23703) show 100% homology with the amino acid sequences of the same receptors of the dog.

The immunogen used to obtain the anti-TRPA1 antibody was the peptide EKQHELIKLIIQKME corresponding to amino acids 1,070–1,085 of rat TRPA1. The homology between the full amino acid sequences of the dog and rat TRPA1 was 82.29%, and correspondence with the specific sequence of the immunogen was 100%.

To identify the vanilloid receptor TRPV1, an antibody recently tested on the canine nervous system (45) was used. The immunogen of the rabbit anti-TRPV1 (ACC-030) was the peptide (C)EDAEVFK DSMVPGEK (824–838) of rat TRPV1. The homology between the specific amino acid sequences of the dog and the rat immunogens was 87.51% and the correspondence with the specific sequence of the immunogen was 75%. In the current study, the rabbit anti-TRPV1 antibody was preincubated with TRPV1 Blocking Peptide (#BLP-CC030; Alomone). After the preasdorption test, no labeling was observed in the keratinocytes and dermal cells (Supplementary Figures 1a–c).

The anti-5-HT1aR antibody was raised using, as an immunogen, a synthetic peptide corresponding to amino acids 100–200 (conjugated to keyhole limpet haemocyanin) of rat 5-HT1aR. The homology between the full amino acid sequences of dog and rat 5HT1aR was 91.94%, and correspondence with the specific sequence of the immunogen was 100%.

The homologies of the receptors studied (CB1R, CB2R, GPR55, PPARα, TRPV1, TRPA1, and 5-HT1aR in the dogs were verified using the “alignment” tool available on the Uniprot database (www.uniprot.org) and the BLAST tool of the National Center for Biotechnology information (NCBI) (www.ncbi.nlm.nih.gov).

### Specificity of the Secondary Antibody

The specificity of the secondary antibody was tested by applying it after omission of the primary antibodies. No stained cells were detected after omitting the primary antibodies.

### Quantitative Analysis

Quantitative analysis of the intensity of the expression of cannabinoid and cannabinoid-related receptors in the keratinocytes of the suprabasal layer was carried out on 7 CTRL- and 8 AD-dogs. For each animal, and each receptor, three randomly selected images of the epidermis were acquired (high magnification, ×400), using the same exposure time for all the images. In each image the signal intensity was analyzed using ImageJ software (Image J, version 1.52t, National Institutes of Health, Bethesda, MD, USA) by standardized thresholds for brightness and contrast were determined empirically and applied to all images. The signal intensity was finally obtained using the Color histogram (gMEAN) tool of the software.

### Statistical Methods

For each receptor the mean of the three values/case of signal intensity in the 7 CTRL- vs. the 8 AD-dogs were compared. Statistical analysis was carried out using GraphPad Prism software (version 8.3, La Jolla, CA). The normality distribution of the data was assessed using the Shapiro-Wilk test. The data normally distributed were analyzed using the Student *T*-test; conversely, the data not normally distributed were analyzed using the Mann-Whitney test. Bonferroni correction to adjust probability values was applied (considering 7 comparisons, Bonferroni corrected *P* is 0.0071). A *P*-value ≤ 0.0071 was considered significant. Because the two sites of skin sampling were groin and axilla in all dogs, a preliminar test to rule out differences of expression was conducted comparing the data of the two sampling sites in the control dogs (Supplementary Figure 2). No difference in receptors expression was apparent in the skin of the control dogs so pooled data of the two sites were used to compare CTRL vs. AD dogs.

## Results

### Histopathology

Normal skin samples did not show inflammatory infiltrates or other pathological changes. The epidermis was thin, and it was composed of three layers of cells: the basal layer (germinal cells), the suprabasal layer (intermediate epithelial cells), generally composed of a couple of cell layers, and the superficial layer (stratum corneum).

All the AD samples had a histopathological diagnosis of chronic hyperplastic mixed perivascular dermatitis, coherent with the inflammatory pattern of canine AD. In particular, the skin showed an epidermis having moderate to severe, focal to diffuse hyperplasia in the superficial (hyperkeratosis) and the suprabasal (acanthosis) layers while, in the dermis, it showed perivascular to interstitial superficial mixed inflammatory infiltrates (lymphocytes, histiocytes, mast cells, plasma cells and few eosinophils) associated with periannessial mixed inflammatory infiltrates in three of the eight cases and apocrine sweat gland dilation in two of the eight cases.

### Cannabinoid and Cannabinoid-Related Receptor Immunolabelling in Keratinocytes

All the receptors studied showed some degree of immunoreactivity (IR) in the basal and suprabasal layers whereas none of the receptors studied were expressed in the *stratum corneum*. Some of the receptors studied were also expressed by dermal cells, hair follicles and dermal glands; however, in the current study, only the immunolabelling expressed by the keratinocytes was considered.

#### CB1R

**Figure 1:**
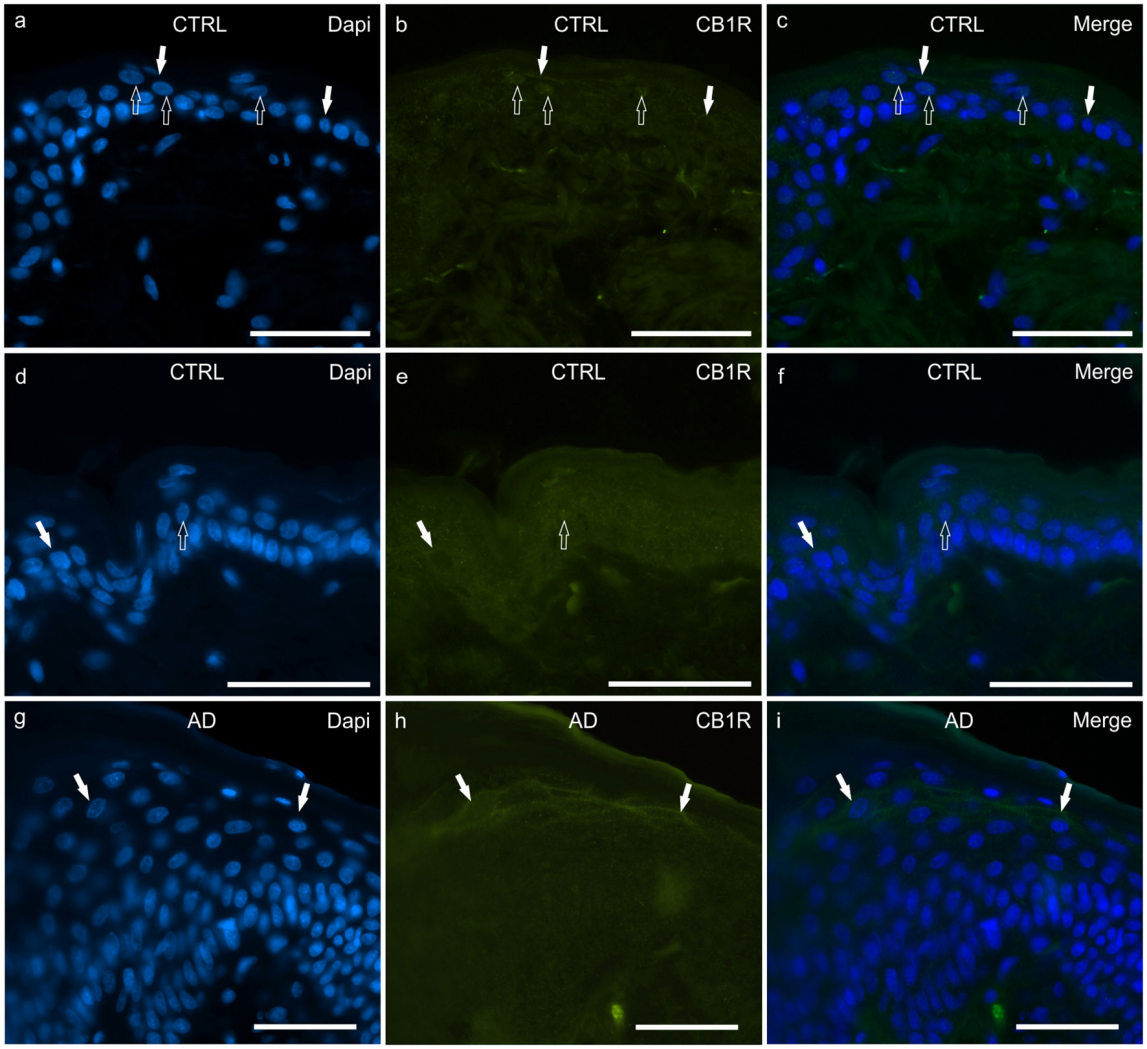
Photomicrographs of cryosections of canine skin showing cannabinoid receptor 1 (CB1R) immunoreactivity (IR) in the tissues of the healthy dogs (CTRL) **(a–f)** and in the dogs with atopic dermatitis (AD) **(g–i)**. Two different anti-CB1R antibodies were used: **(a–c)** Rabbit anti CB1R (Orb10430) antibody; **(d–f)** Rabbit anti CB1R (ab23703) antibody. **(a–f)** The open arrows indicate DAPI-labeled nuclei of the epithelial cells which displayed faint CB1R-IR; the white arrows indicate faint CB1R-IR of the cell membrane of the keratinocytes. **(g–i)** In the AD-dogs, the keratinocytes of the upper layers (in proximity to the *stratum corneum*) showed moderate CB1R-IR of their cell membranes (white arrows). **(g–i)** were obtained using the Orb10430 antibody. Bar, 50μm.

In the CTRL-dogs, both the anti-CB1R antibodies utilized in the current study identified very faint and granular CB1R-IR in the cytoplasm of the basal and suprabasal keratinocytes. Both antibodies also identified CB1R-IR at the level of the cell membranes and nuclei of a few keratinocytes (Figures 1a–f).

In the AD-dogs, CB1R-IR showed the same pattern of immunolabelling observed in the CTRL-dogs; however, using both anti-CB1R antibodies, the cells of the intermediate layer, located just beneath the *stratum corneum*, showed moderate-to-bright CB1R-IR of the cell membrane (Figures 1g–i). Only the anti-CB1R antibody Orb10430 identified moderate and granular CB1R-IR in the nuclei of keratinocytes (data not shown). In the epidermis of the AD-dogs, CB1R-IR appeared slightly increased; however, it was not statistically significant (*P* = 0.2432) (**Figure 8a**).

#### CB2R

**Figure 2:**
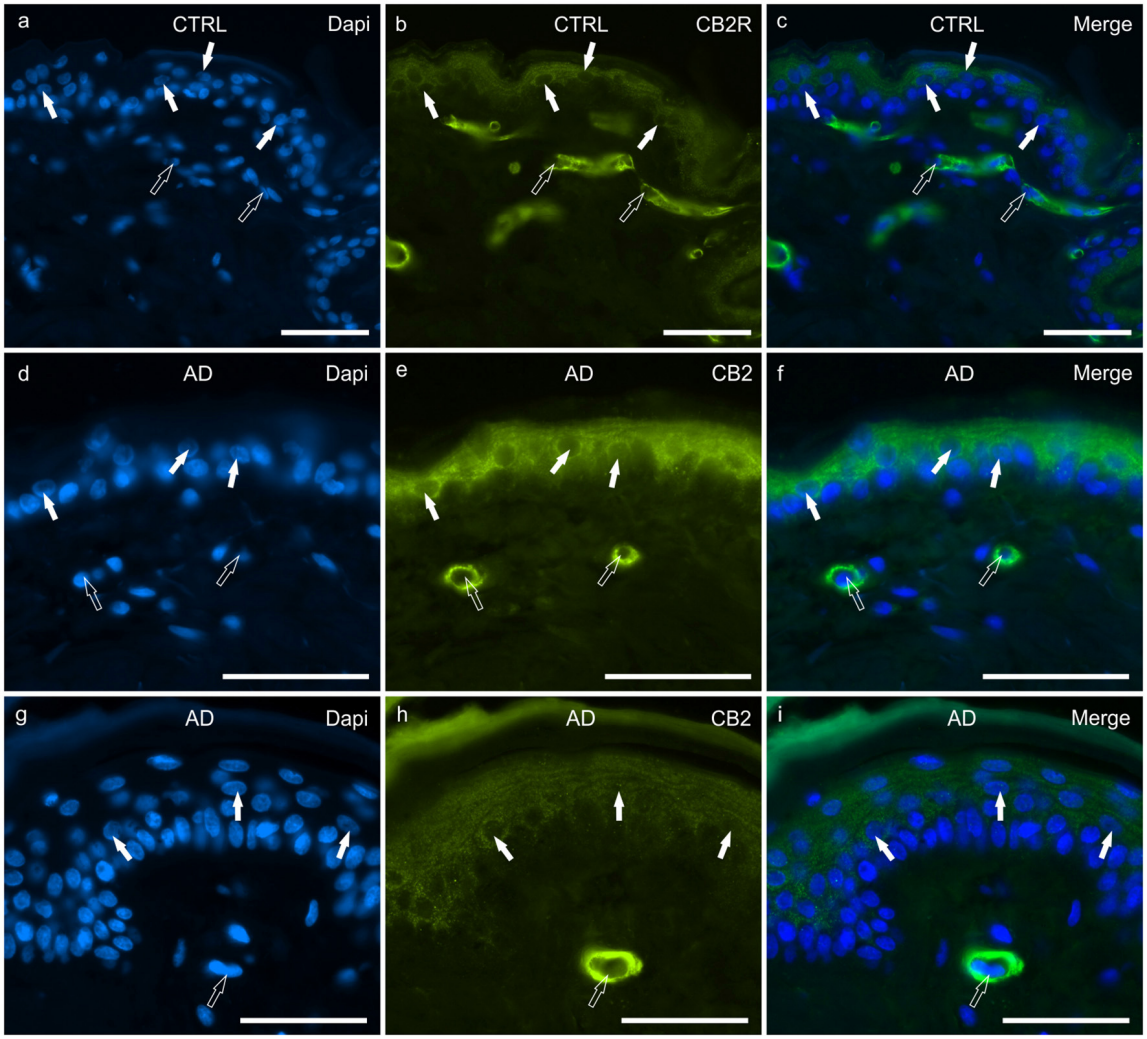
Photomicrographs of cryosections of canine skin showing cannabinoid receptor 2 (CB2R) immunoreactivity (IR) in the tissues of the healthy dogs (CTRL) **(a–c)** and in the dogs with atopic dermatitis (AD) **(d–i)**. **(a–c)** The white arrows indicate the DAPI-labeled nuclei of the keratinocytes showing moderate CB2R-IR of the cytoplasm and cell membrane. The open arrows indicate the DAPI-labeled nuclei of vascular endothelial cells of the dermis showing bright CB2R-IR. **(d–i)** The white arrows indicate the DAPI-labeled nuclei of the keratinocytes of the dogs with AD showing bright CB2R-IR. The open arrows indicate the DAPI-labeled nuclei of the vascular endothelial cells of the dermis showing bright CB2R-IR. Bar, 50 μm.

In the CTRL-dogs, granular CB2R-IR was expressed by the cytoplasm and the cell membrane of keratinocytes. In particular, CB2R-IR was faint in the cells of the basal layer whereas it was moderate in the cells of the suprabasal layer (Figures 2a–c). The endothelial cells of the subepidermal blood capillaries showed bright CB2R-IR (Figures 2a–c).

In the AD-dogs, CB2R-IR was faint-to-moderate in the cytoplasm and the cell membrane of keratinocytes of the basal layer and bright in the cells of the suprabasal layer (Figures 2d–i). The subepidermal blood capillaries were generally more numerous and larger in the AD-dogs, and showed bright CB2R-IR (Figures 2d–i). Statistical analysis confirmed that, in the AD vs. the CTRL dogs, there was a significant increase in the CB2R signal in terms of intensity (*P* = 0.0022, significant even after Bonferroni correction) (**Figure 8b**).

#### GPR55

**Figure 3:**
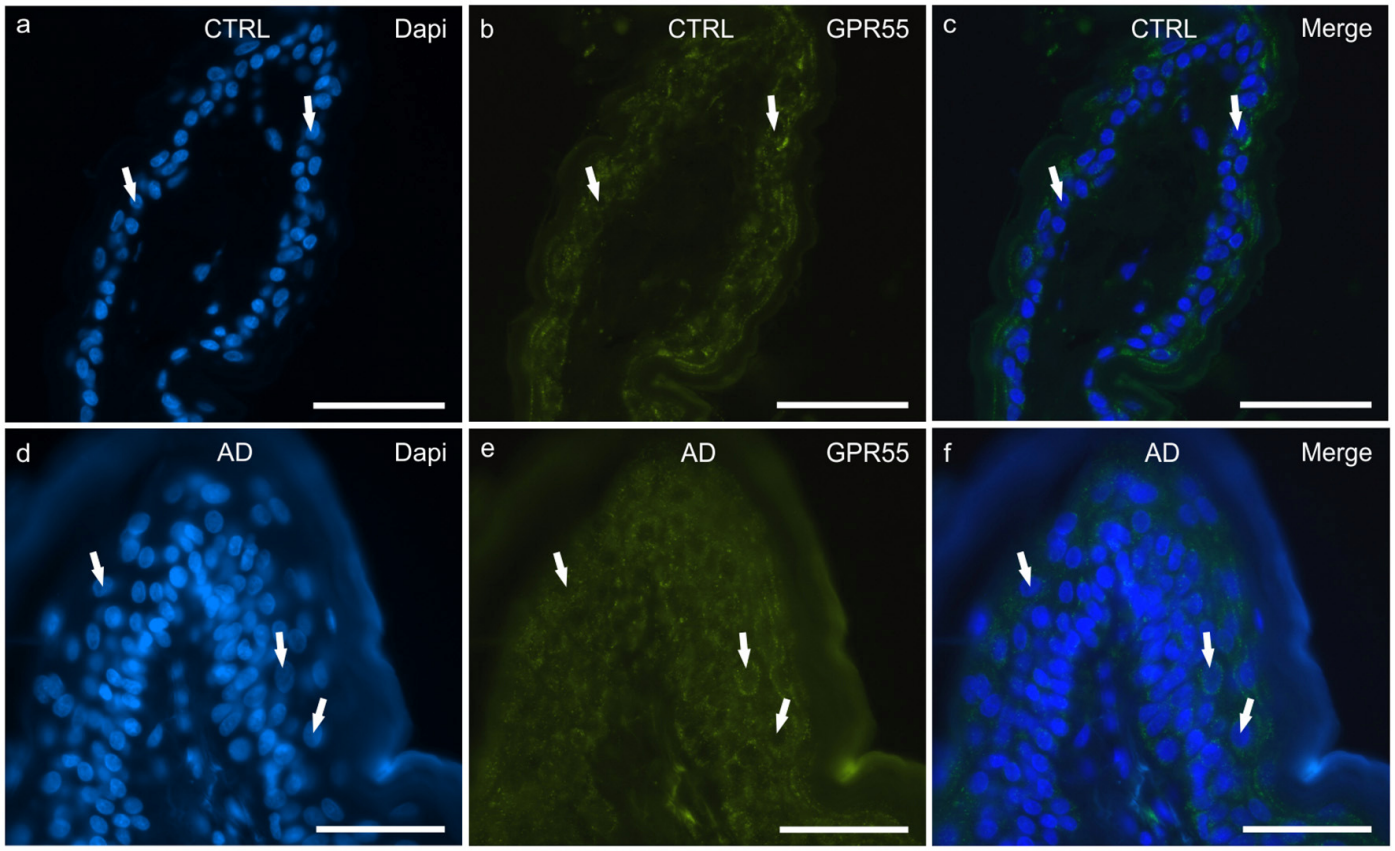
Photomicrographs of cryosections of canine skin showing G protein-coupled receptor 55 (GPR55) immunoreactivity (IR) in the tissues of the healthy dogs (CTRL) **(a–c)** and in the dogs with atopic dermatitis (AD) **(d–f)**. **(a–c)** The white arrows indicate the DAPI-labeled nuclei of the keratinocytes showing moderate cytoplasmic GPR55-IR. **(d–f)** The white arrows indicate some DAPI-labeled nuclei of the cells of the suprabasal layer showing moderate-to-bright GPR55-IR. Bar, 50 μm.

In the CTRL-dogs, moderate-to-bright granular cytoplasmic GPR55-IR was expressed in the cells of the basal and suprabasal layers of the epidermal cells (Figures 3a–c).

In the AD-dogs, bright and granular GPR55-IR was expressed in the cytoplasm of the proliferative cells of the basal layer (Figures 3d–f). In the suprabasal layer, moderate GPR55-IR was diffusely expressed by the cytoplasm of the keratinocytes. In the epidermis of the AD-dogs, GPR55-IR appeared slightly increased; however, it was not statistically significant (*P* = 0.3191) (**Figure 8c**).

#### PPARα

**Figure 4:**
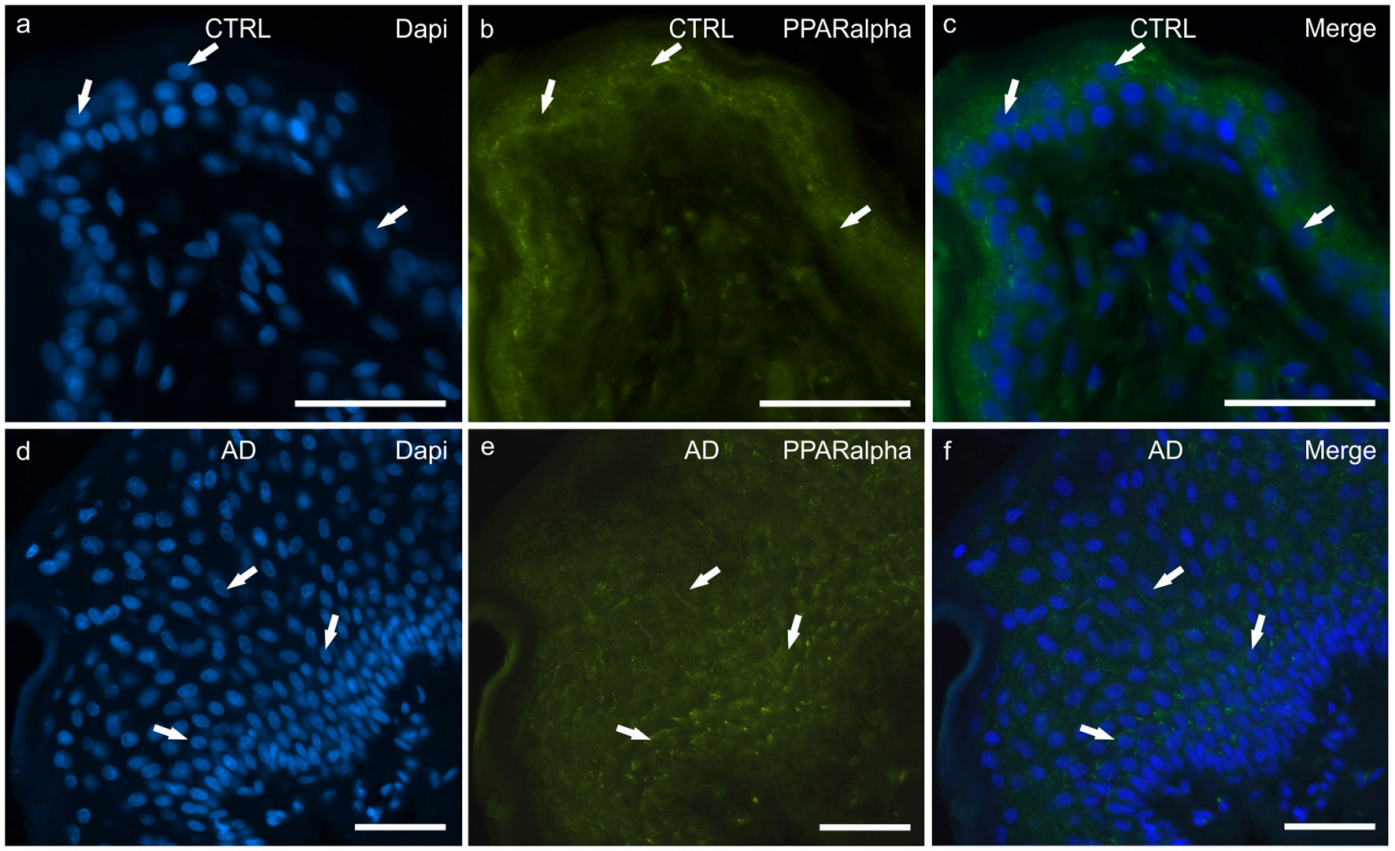
Photomicrographs of cryosections of canine skin showing peroxisome proliferator-activated receptors alpha (PPARα) immunoreactivity (IR) in the tissues of the healthy dogs (CTRL) **(a–c)** and in the dogs with atopic dermatitis (AD) **(d–f)**. **(a–c)** The arrows indicate the DAPI-labeled nuclei of some keratinocytes showing moderate cytoplasmic PPARα-IR. **(d–f)** The arrows indicate the DAPI-labeled nuclei of some keratinocytes showing moderate cytoplasmic PPARα-IR in the cells of the basal and the suprabasal layers. Bar, 50 μm.

In the CTRL-dogs, faint and moderate PPARα-IR was expressed in the cytoplasm of the keratinocytes of the basal and the suprabasal layers, respectively; the cells of the suprabasal layer also showed bright PPARα-IR of the cell membrane (Figures 4a–c).

In the AD-dogs, all the cells of the basal and the suprabasal layers expressed moderate-to-bright granular PPARα-IR (Figures 4d–f). In the epidermis of the AD-dogs, PPARα-IR appeared slightly increased; however, it was not statistically significant (*P* = 0.0728) (**Figure 8d**).

#### TRPV1

**Figure 5:**
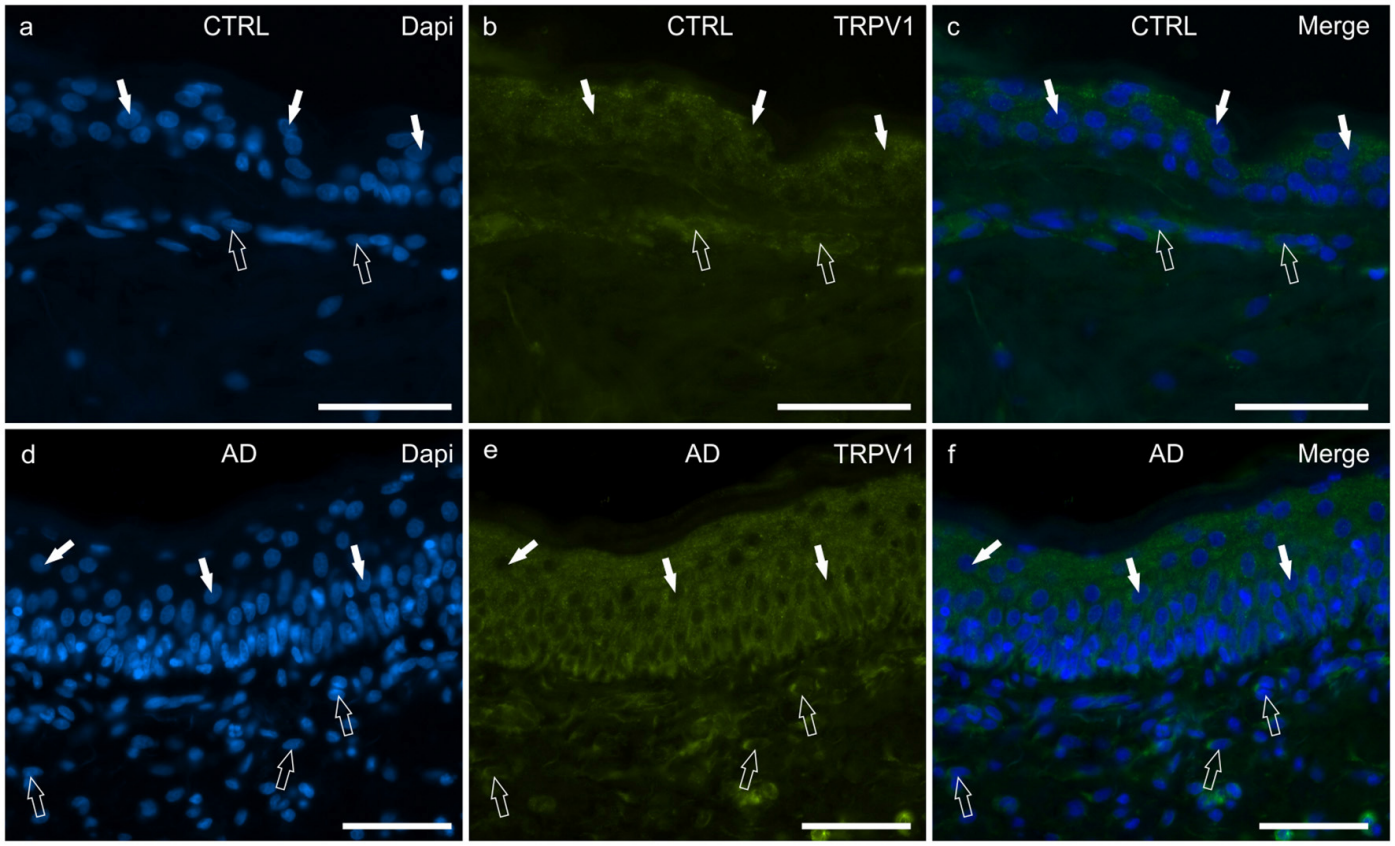
Photomicrographs of cryosections of canine skin showing transient receptor potential vanilloid 1 (TRPV1) immunoreactivity (IR) in the tissues of the healthy dogs (CTRL) **(a–c)** and in the dogs with atopic dermatitis (AD) **(d–f)**. **(a–c)** The white arrows indicate some DAPI-labeled nuclei of the keratinocytes of the CTRL dogs showing moderate cytoplasmic TRPV1-IR. The open arrows indicate some DAPI-labeled nuclei of the dermal cells showing moderate TRPV1-IR. **(d–f)** The white arrows indicate some DAPI-labeled nuclei of the keratinocytes of the AD dogs showing bright cytoplasmic TRPV1-IR. The open arrows indicate some DAPI-labeled nuclei of the dermal cells showing bright TRPV1-IR. Bar, 50 μm.

In the CTRL-dogs, the cytoplasm of the cells of the basal and the suprabasal layers expressed faint-to-moderate cytoplasmic TRPV1-IR (Figures 5a–c).

In the AD-dogs, TRPV1-IR was moderately expressed by the cells of the basal layer. The cells of the intermediate layer expressed moderate-to-bright TRPV1-IR, especially in the cells located close to the *stratum corneum* (Figures 5d–f). In the epidermis of AD-dogs, TRPV1-IR appeared increased; hovewer, it was not statistically significant (*P* = 0.0252) (**Figure 8e**).

#### TRPA1

**Figure 6:**
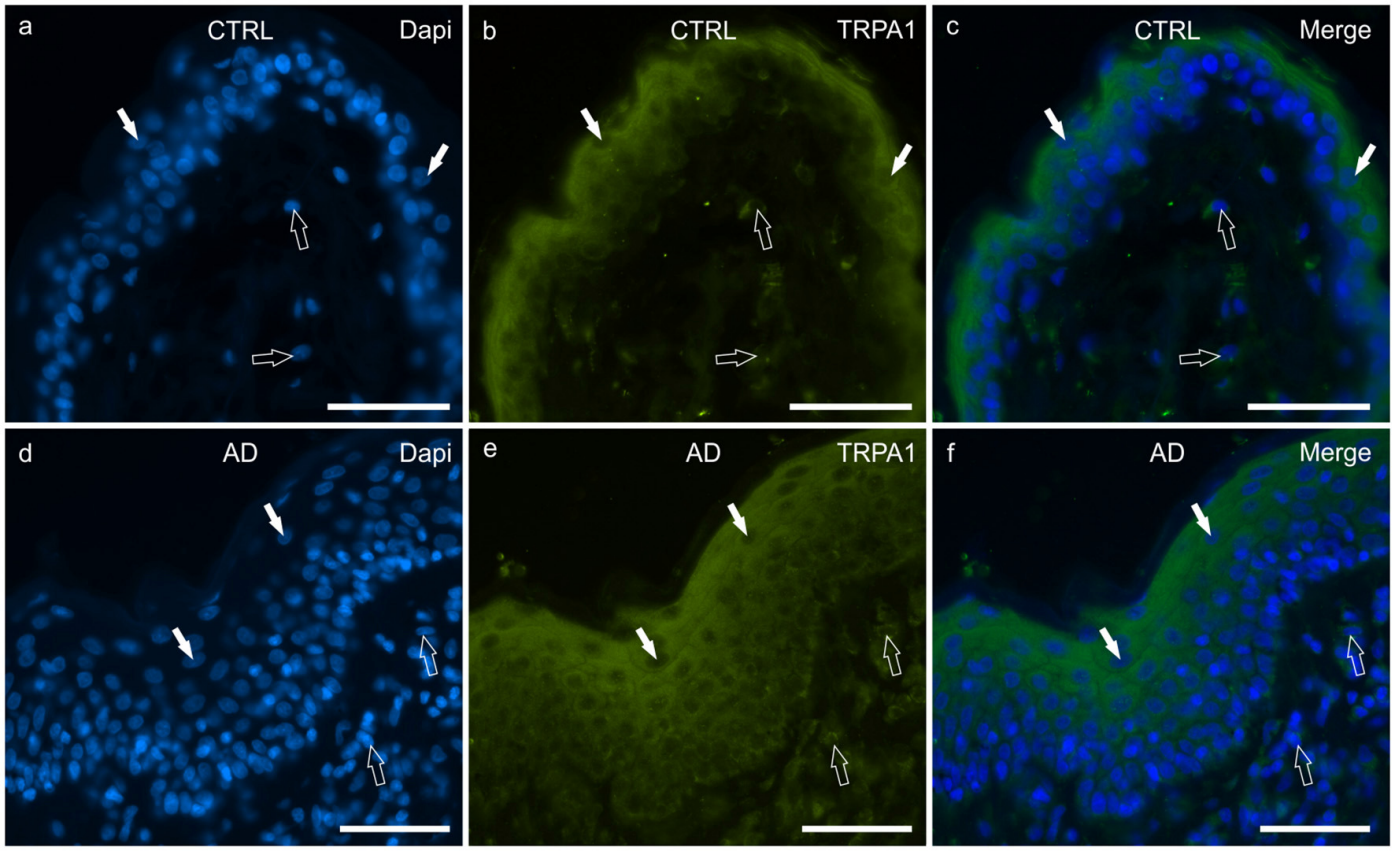
Photomicrographs of cryosections of canine skin showing transient receptor potential ankyrin 1 (TRPA1) immunoreactivity (IR) in the tissues of the healthy dogs (CTRL) **(a–c)** and in the dogs with atopic dermatitis (AD) **(d–f)**. **(a–c)** The white arrows indicate some DAPI-labeled nuclei of the keratinocytes of the CTRL dogs showing moderate cytoplasmic TRPV1-IR. The TRPA1 immunolabelling was brighter in the more superficial cells. The open arrows indicate some DAPI-labeled nuclei of the dermal cells showing moderate TRPV1-IR. **(d–f)** The white arrows indicate some DAPI-labeled nuclei of the keratinocytes of the AD dogs showing bright cytoplasmic TRPV1-IR. Not all the cells of the suprabasal layer showed the same degree of TRPA1-IR which was brighter in the cytoplasm of the more superficial cells. The open arrows indicate some DAPI-labeled nuclei of the dermal cells showing bright TRPA1-IR. Bar, 50 μm.

In the CTRL-dogs, faint cytoplasmic TRPA1-IR was expressed by the cells of the basal layer whereas the cells of the suprabasal layer expressed moderate-to-bright TRPA1-IR (Figures 6a–c).

In the AD-dogs, the TRPA1-IR was faint in the cytoplasm of the basal layer cells and in the deepest cells of the suprabasal layer; the keratinocytes in the upper portion of the suprabasal layer expressed moderate-to-bright cytoplasmic TRPA1-IR (Figures 6d–f). Statistical analysis confirmed that, in the AD vs. the CTRL dogs, there was a significant increase in the TRPA1-IR signal in terms of intensity (*P* = 0.0034, significant even after Bonferroni correction) (**Figure 8f**).

#### 5-HT1aR

**Figure 7:**
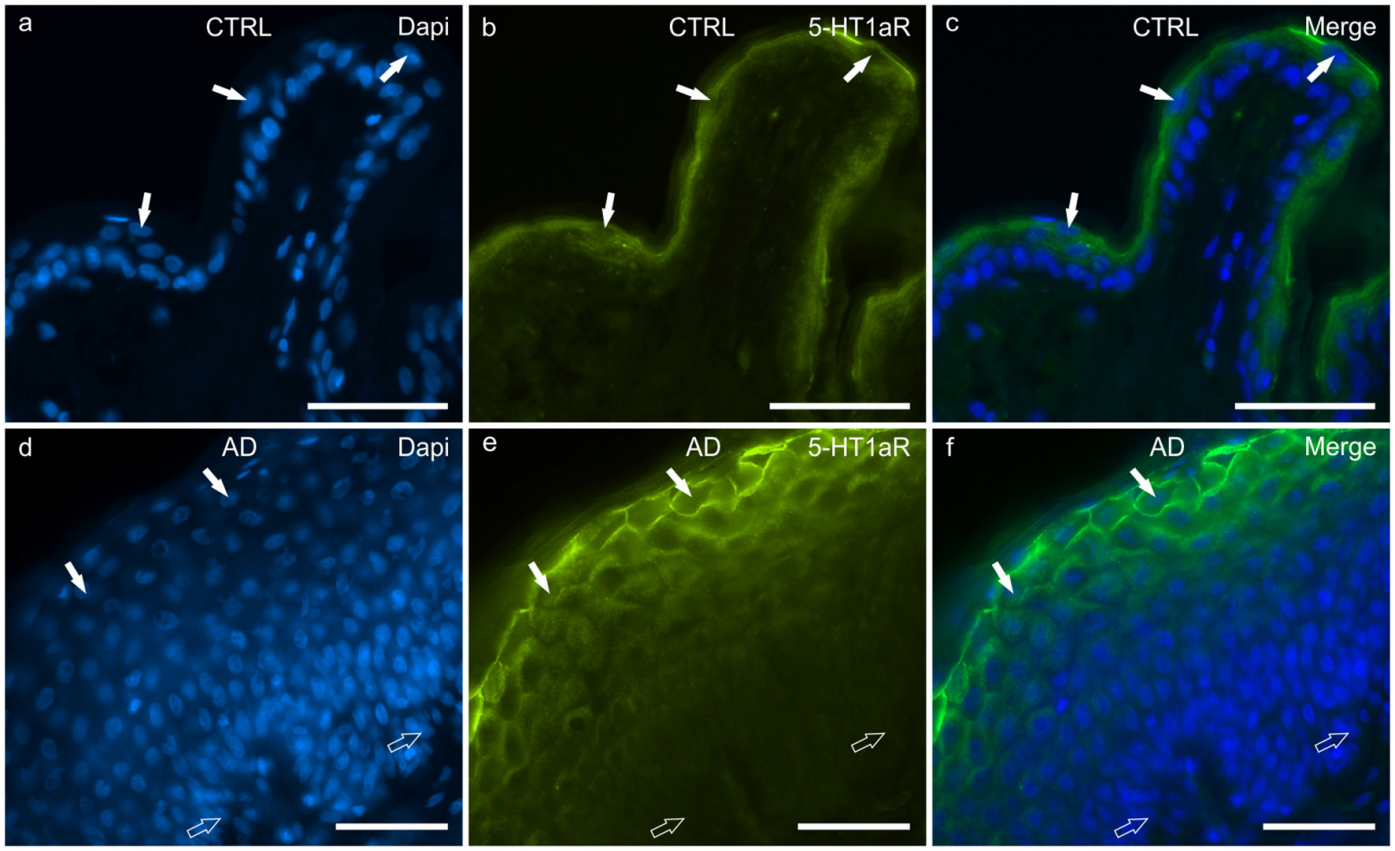
Photomicrographs of cryosections of canine skin showing showing serotonin 1a receptor (5HT1aR) immunoreactivity (IR) in the tissues of the healthy dogs (CTRL) **(a–c)** and in the dogs with atopic dermatitis (AD) **(d–f)**. **(a–c)** The white arrows indicate the DAPI-labeled nuclei of some keratinocytes of the suprabasal layer showing bright 5-HT1aR-IR. **(d–f)** In the AD dogs, the cell membrane of the more superficial keratinocytes showed very bright 5-HT1aR-IR whereas the germinative cells of the basal layer and the deepest cells of the suprabasal layer were either nearly 5-HT1aR negative or expressed very faint 5-HT1aR-IR. Bar, 50 μm.

In the CTRL-dogs, the cells of the basal layer were nearly 5-HT1aR negative or were faintly immunolabelled. In the suprabasal layer, moderate and bright 5-HT1aR-IR was expressed by the cytoplasm and the cell membranes of the keratinocytes, respectively (Figures 7a–c).

**Figure 8:**
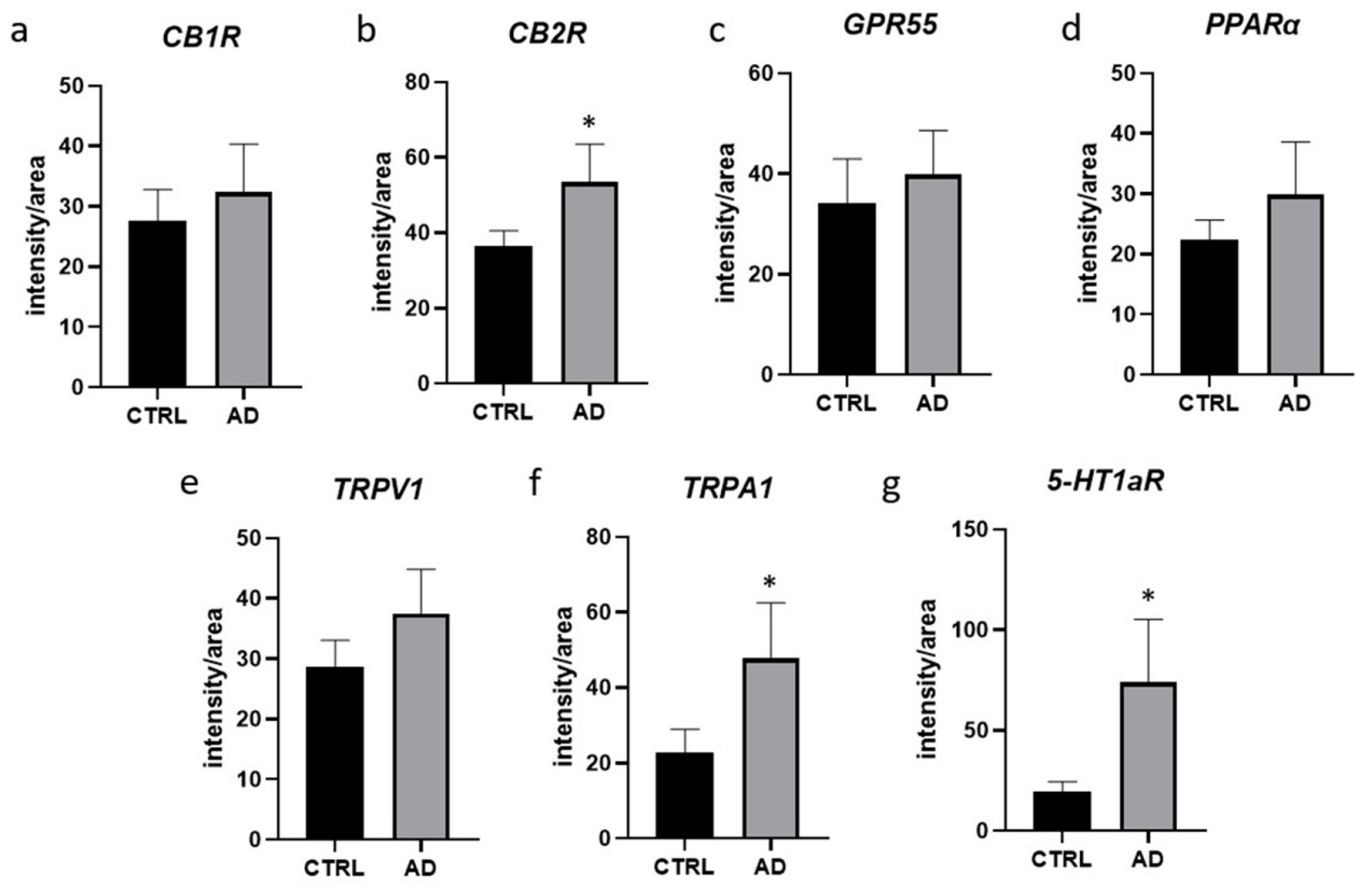
Quantification of the intensity of the expression of CB1R **(a)**, CB2R **(b)**, GPR55 **(c)**, PPARα **(d)**, TRPV1 **(e)**, TRPA1 **(f)**, 5-HT1aR **(g)**, in the suprabasal layers of 7 CTRL- and 8 AD-dogs. Data are represented as Mean ± SD and were analyzed using the Student *T*-test. *P* < 0.0071 (*).

In the AD-dogs, the cells of the basal layer were almost 5-HT1aR negative. The cells of the suprabasal layer showed an increasing gradient of granular cytoplasmic 5-HT1aR-IR which was also brightly expressed by the cell membrane of the keratinocytes adjacent to the *stratum corneum* (Figures 7d–f). In the epidermis of the AD-dogs, 5-HT1aR-IR was significantly upregulated (*P* = 0.0015, significant even after Bonferroni correction) (Figure 8g).

## Discussion

### Cannabinoid Receptors in Keratinocytes

Cannabinoid receptors have been localized in the nervous system as well as on cells of the immune system (44–46). However, a growing body of evidence has indicated that CB1R and CB2R are also expressed by the keratinocytes of humans (46, 47) and mice (47), and that cannabinoids are important mediators in the skin (37). Studies involving humans have shown that endocannabinoid AEA inhibits the formation of cornified envelopes, a hallmark of keratinocyte differentiation, by means of the activation of the CB1R (30); it inhibits proliferation and reduces the differentiation of epidermal keratinocytes (48). A study on mice has shown that the CB1R is functionally expressed by keratinocytes and helps to limit the secretion of the proinflammatory chemokines which regulate T cell-dependent inflammation during contact hypersensitivity (49). Mice lacking the CB1R, only in the keratinocytes, showed increased Th2-type allergic skin inflammatory responses and delayed epidermal barrier repair (50). In the current study, CB1R-IR was not different in the AD-dogs and the healthy dogs. The present findings were consistent with those of Campora et al. (40).

Cannabinoid receptor 2 has been found in the human (46), mouse (51) and dog epidermis (40), suggesting that CB2R could play a role during the differentiation of keratinocytes.

An interesting study involving rats showed that the CB2R is also involved in the regulation of skin pain; in fact, CB2R activation stimulates the release of beta-endorphins (endogenous opioid) from keratinocytes which modulate nociception by acting on the opioid receptors on the primary sensory fibers of the skin (52).

### Cannabinoid-Related Receptors in Keratinocytes

As with other cutaneous diseases, not all of the anti-inflammatory effects of cannabinoids depend upon CB1R and CB2R activation (49). Anandamide, for instance, also activates GPR55 (53), PPARs (54) and TRPV1 (55) while PEA exerts its anti-inflammatory effect on contact dermatitis acting on the TRP channels rather than on the CB receptors (56).

Little is known regarding the physio-pathological relevance of the orphan GPR55 in the skin where the receptor is expressed in keratinocytes and melanocytes, and is activated by several cannabinoids to regulate skin homeostasis (56). The findings of the present study, showing GPR55-IR in the dog skin, is consistent with that reported for mice (57). Studies involving humans and mice have shown that GPR55 promotes skin tumor development by enhancing invasiveness and tumorigenicity (57, 58). In the current study, GPR55-IR was mainly expressed by the germinative cells of the basal layer of the AD-dogs; this evidence suggested the role of the GPR55 receptor in keratinocyte proliferation. Cannabidiol, which acts on GPR55 as an antagonist (59), could potentially regulate skin physiology due to its antiproliferative and anti-inflammatory properties, and its ability of modulating keratinocyte differentiation (60).

Peroxisome proliferator-activated receptors (PPARα, PPARβ/δ and PPARγ) play a role in skin homeostasis; in fact, PPARs modulate a wide variety of skin functions, including keratinocyte proliferation, epidermal barrier formation, wound healing, melanocyte proliferation and sebum production (61, 62). In the skin of mice, PPARα is located in the epidermal nuclei of the basal and suprabasal cells (63) whereas, in human skin, PPARγ is located in the basal layer of the epidermis (64). In the present study, PPARα was identified in the keratinocytes of the basal and the suprabasal layers of the skin of both the CTRL- and the AD-dogs. Since it has been shown that the predominat PPAR subtype in the keratinocytes of humans are PPARβ/δ (62), it cannot be excluded that, in dogs, the predominant PPARs are PPARα. However, the anti-PPARβ/δ and the anti-PPARγ antibodies were not used in the current study.

It has been shown that TRP receptors are present in various types of skin cells and are involved in different functions, such as the formation and maintenance of the skin barrier, cell differentiation, cell growth, temperature perception, and immunological and inflammatory reactions (65, 66). The TRP channels are expected to play a new role in the pruritus pathogenesis due to the assumption that the itching sensation could be significantly affected by temperature (67).

The vanilloid receptor 1 (VR1 or TRPV1) is not only expressed in the sensory neurons (45, 68), but also on the keratinocytes and DCs in the skin (7, 69–71). The expression of TRPV1 in keratinocytes could play a role in the inflammation which occurs as a result of epidermal damage or insult, and may therefore function as a sensor for noxious cutaneous stimulation. The activation of the epidermal TRPV1 by capsaicin resulted in the increased release of IL-8 and prostaglandin E2, which was attenuated by a TRPV1 antagonist (capsazepine) (72). In human AD patients, it has been reported that the amount of IL-8, a potent chemoattractant for T cells and neutrophils in the *stratum corneum*, is related to the severity of local skin inflammation (73). In canine keratinocytes, IL-8 production is enhanced under allergic skin conditions (74). While TRPV1 antagonists could play a potential role in the treatment of humans AD (71, 75), nothing can be said about dog AD yet, also given that in the current study the keratinocytes of the AD-dogs did not show significant upregulation of TRPV1-IR in the suprabasal layer cells when compared to the CTRL-dogs.

In AD-like murine models, a TRPV1 antagonist (PAC-14028) was seen to show antipruritic effects, to improve skin barrier functions and to suppress the allergic inflammation by means of the modulation of epidermal differentiation markers which blocked the secretion of neuropeptides and suppressed Th2 cytokines (75–77). Cannabidiol, which stimulates and desensitizes TRPV1 channels (78, 79), inhibits the production of proinflammatory cytokines (i.e. IL-6, IL-8 and TNF-a) and of the monocyte chemotactic protein-2 (MCP-2) chemokine in stimulated human keratinocyte cells (HaCaT cells) (80). In addition, CBD increases AEA levels in stimulated HaCaT cells, which reduce IL-8, IL-6 and MCP-2 protein levels (80).

Another TRP channel with a major role in itch transmission is TRPA1 which is expressed in sensory nerve fibers and is stimulated by cold temperatures (≤ 17°C) (81) and by numerous natural compounds, including pungent skin irritants and enviromental irritants (82). In the skin of mice, it has been shown that TRPV1/TRPA1 sensory nerve fibers also express the receptor for IL-31 and establish a critical neuroimmune link between Th2 cells and sensory nerves for the generation of T cell-mediated itch (20, 63, 64). As seen for TRPV1, TRPA1 is also expressed in non-neuronal cells, such as the epithelial cells of the skin in which it seems to be critically involved in a series of physiological skin functions, including the formation and maintenance of physico-chemical skin barriers, skin cells, and tissue growth and differentiation (83). Atoyan et al. (84) provided evidence that “ice-cold” TRPA1, like its “warm and hot” counterpart TRPV1, is broadly expressed in the non-neuronal cells of human skin and could be directly involved in the regulation of keratinocyte proliferation (82). In human skin, TRPA1-IR was detected in the keratinocytes and melanocytes of the epidermis (84, 85).

In keratinocytes from skin lesions of human AD patients, TRPA1-IR was increased (86). In the current study, keratinocytes of the AD-dogs showed a significant upregulation of TRPA1-IR.

It seems evident that TRPA1 not only serves as a sensor for pruritogens, but is also essential for maintaining skin inflammation, as shown in AD and contact dermatitis models in which treatment with TRPA1 inhibitors reduces skin swelling, epidermal water loss and leukocyte infiltration (87–89).

*S*erotonin (5-HT) plays an essential role in the skin, acting as a mediator between this organ and the neuroendocrine system; 5-HT and its receptors play an important role in the regulation of immune signaling (90). In the human skin, 5-HT is stored in the MCs and is released upon immunoglobulin E (IgE) cross-linking (91). The expression of genes coding for 5-HT1aR has been demonstrated in human keratinocytes and melanocytes (92). In human patients with AD, it has been shown that keratinocytes and DCs express 5-HT1aR (93).

In the current study, 5-HT1aR-IR was observed in the suprabasal layer of the epidermis of the CTRL-dogs; this finding was consistent with that of Lundeberg et al. (94) who observed the same distribution pattern of 5-HT1aR-IR in the human skin. The presence of 5-HT1aR-IR in the suprabasal layer of the epidermis of dogs could indicate a role of the receptor in the keratinocytes differentiation, as has been suggested in humans (95). A recent study showed that the 5-HT1aR plays an important role during the healing of the skin (96). In AD-dogs, significant upregulation of 5-HT1aR-IR was observed; this finding appears to be in contrast with that reported by Slominski et al. (97) who indicated that the levels of 5-HT1aR could decrease in inflammatory skin diseases.

Drugs, such as 5-HT1aR agonists, are useful in the clinical management of the stress-associated aggravation of AD. Multitargeted mechanisms might underlie the clinical efficacy of such drugs. In fact, the favorable effect of 5-HT1aR agonists could play a central role by improving the psychological status of patients with AD. However, 5-HT1aR agonists might also have a therapeutic action on the homeostasis of the epidermal cells and on the inhibition of stress-induced MCs degranulation (MC express 5-HT1aR immunoreactivity as well) (98).

### Limitations

The limited number of dogs considered represents a limitation of the present study. Furthermore, no markers for cells residing between the keratinocytes, such as melanocytes, Langerhans cells, immune cells and inflammatory cells, were used. Additional biomolecular studies are needed to strengthen the data obtained in this study. In addition, the expression of the receptors in hair follicles, sebaceous glands, blood vessels, and inflammatory cells has not been reported as they will be documented in a future paper. However, preliminary results indicated that MCs and T cells showed CB2- and GPR55-IR.

## Conclusion

Immunoreactivity for the canonical cannabinoid receptors (CB1R and CB2R) and the cannabinoid-related receptors (GPR55, TRPV1, TRPA1, PPARα, and 5-HT1aR) has been observed in the keratinocytes of healthy dogs and dogs with AD. The presence of these receptors in healthy keratinocytes supports the hypothesis that the ECS in dogs plays a role in the maintenance of skin homeostasis, contributing to the regulation of the renewal and differentiation of epithelial cells, and of barrier permeability. Considering the general upregulation of the target receptors of the skin epithelium of the AD-dogs, with significant values for CB2R, TRPA1, and 5-HT1aR, it seems that the ECS could represent an interesting potential therapeutic target. Therefore, it is reasonable to consider that endocannabinoids and *Cannabis*-derived molecules could play a role in counteracting the skin barrier dysregulation during AD. Studies carried out mainly on laboratory animals have indicated that CBD, unlike Δ9-THC, does not produce psychotropic effects and may exert beneficial effects on pain perception (26, 33); in addition, CBD shows anti-inflammatory and anti-anxiety properties (99, 100) which can improve AD symptoms.

## Data Availability Statement

The raw data supporting the conclusions of this article will be made available by the authors, without undue reservation.

## Ethics Statement

All procedures were approved by the National Health Authority (No. 1303/2021) in accordance with DL 26/2014 and European Union Directive 2010/63/EU, under the supervision of the Central Veterinary Service of the University of Bologna. Written informed consent was obtained from the owners for the participation of their animals in this study.

## Author Contributions

RC, MM, and GS contributed to the study design. FA provided the skin biopsies for the cryosections. MDS, RZC, FG, and CT carried out the immunohistochemical experiments. RC and FG acquisition of data. RC and MM drafting of the manuscript. All authors contributed to execution of the study, interpreted the data, and approved the final manuscript.

## Funding

This study received a grant from ElleVet Sciences (2021).

## Conflict of Interest

The authors declare that the research was conducted in the absence of any commercial or financial relationships that could be construed as a potential conflict of interest.

## Publisher's Note

All claims expressed in this article are solely those of the authors and do not necessarily represent those of their affiliated organizations, or those of the publisher, the editors and the reviewers. Any product that may be evaluated in this article, or claim that may be made by its manufacturer, is not guaranteed or endorsed by the publisher.
