# Supplementary material for: Distribution of Cannabinoid Receptors in Keratinocytes of Healthy Dogs and Dogs With Atopic Dermatitis
Source: Front Vet Sci. 2022 Jul 8;9:915896. doi: 10.3389/fvets.2022.915896 (PMC9305491; doi:10.3389/fvets.2022.915896)
Supplement: Supplementary file 1 [file Data_Sheet_1.PDF]

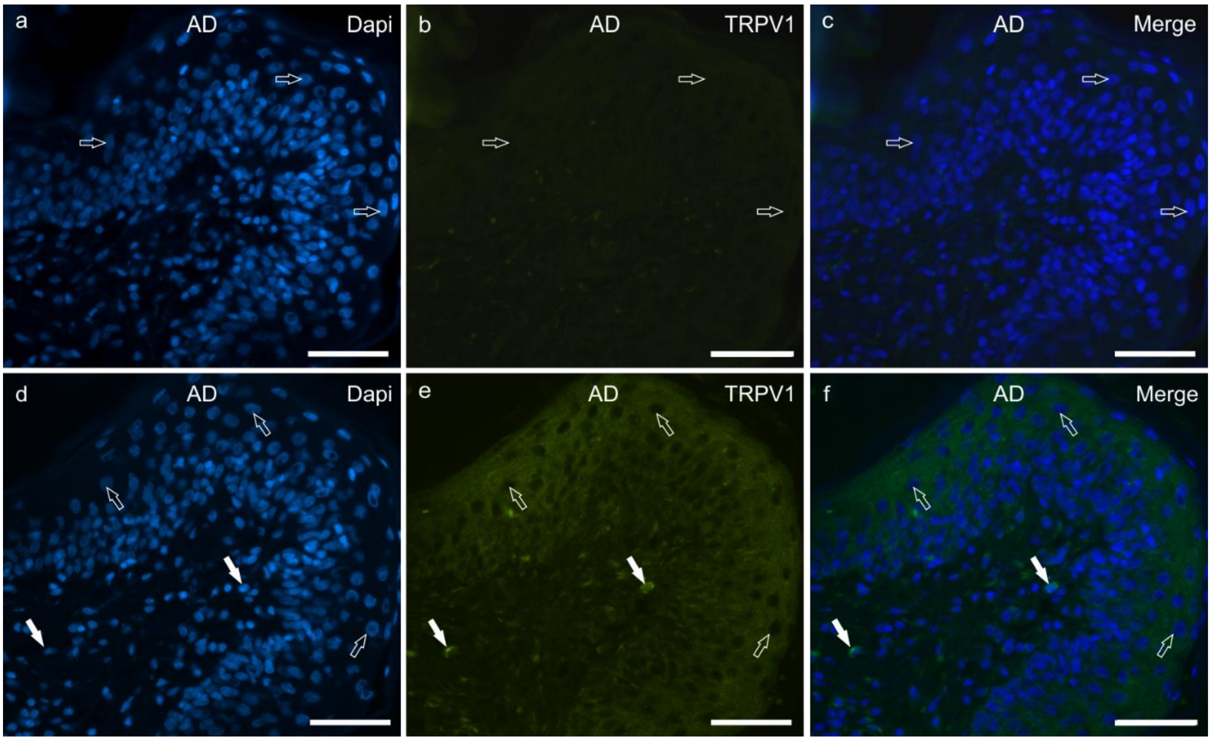

Supplementary Fig. 1. Photomicrographs of two serial cryosections of canine skin on which the polyclonal antibody (ACC-030) against transient receptor potential vanilloid 1 (TRPV1) was applied in combination with the original antigen (blocking peptide; BLP-CC030) which was used for immunization during polyclonal antibody generation (a-c), or alone (d-f). In the section in which the anti-TRPV1 antibody was applied following preincubation with its blocking peptide, the TRPV1-IR was very faint or absent. The open arrows indicate some DAPI-labelled nuclei of the epidermal cells which were TRPV1 negative (d-f). In the section in which the blocking peptide was not applied, the epidermal cells (open arrows) and some dermal cells (white arrows) showed bright TRPV1-IR.

Bar: 50 µm.
