# Supplementary material for: Distribution of Cannabinoid Receptors in Keratinocytes of Healthy Dogs and Dogs With Atopic Dermatitis
Source: Front Vet Sci. 2022 Jul 8;9:915896. doi: 10.3389/fvets.2022.915896 (PMC9305491; doi:10.3389/fvets.2022.915896)
Supplement: Supplementary file 2 [file Data_Sheet_2.PDF]

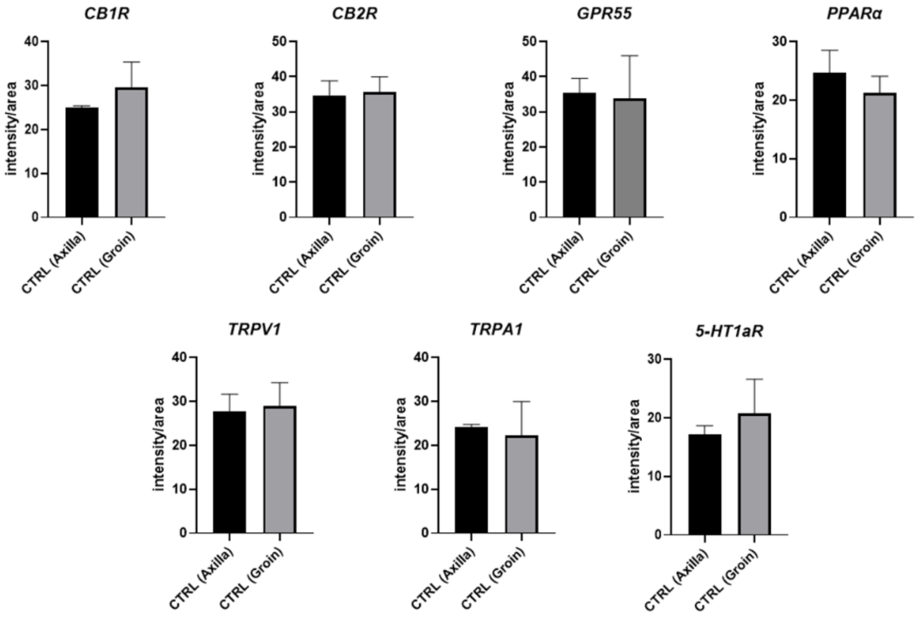

Supplementary Fig. 2. Quantification of the expression of CB1R, CB2R, GPR55, PPARα, TRPV1, TRPA1, and 5-HT1aR of axilla and groin skin samples of control (CTRL) dogs. No differences in immunoreactivity intensity between the two areas were found. Data are represented as Mean ± Standard Deviation and were analyzed using the Mann-Whitney test.
